# III/V-on-Si MQW lasers by using a novel photonic integration method of regrowth on a bonding template

**DOI:** 10.1038/s41377-019-0202-6

**Published:** 2019-10-09

**Authors:** Yingtao Hu, Di Liang, Kunal Mukherjee, Youli Li, Chong Zhang, Geza Kurczveil, Xue Huang, Raymond G. Beausoleil

**Affiliations:** 1 Hewlett Packard Labs, Hewlett Packard Enterprise, 1501 Page Mill Road, Palo Alto, CA 94304 USA; 2 Materials Department, University of California Santa Barbara, Santa Barbara, CA 93106 USA

**Keywords:** Diode lasers, Silicon photonics

## Abstract

Silicon photonics is becoming a mainstream data-transmission solution for next-generation data centers, high-performance computers, and many emerging applications. The inefficiency of light emission in silicon still requires the integration of a III/V laser chip or optical gain materials onto a silicon substrate. A number of integration approaches, including flip-chip bonding, molecule or polymer wafer bonding, and monolithic III/V epitaxy, have been extensively explored in the past decade. Here, we demonstrate a novel photonic integration method of epitaxial regrowth of III/V on a III/V-on-SOI bonding template to realize heterogeneous lasers on silicon. This method decouples the correlated root causes, i.e., lattice, thermal, and domain mismatches, which are all responsible for a large number of detrimental dislocations in the heteroepitaxy process. The grown multi-quantum well vertical p–i–n diode laser structure shows a significantly low dislocation density of 9.5 × 10^4^ cm^−2^, two orders of magnitude lower than the state-of-the-art conventional monolithic growth on Si. This low dislocation density would eliminate defect-induced laser lifetime concerns for practical applications. The fabricated lasers show room-temperature pulsed and continuous-wave lasing at 1.31 μm, with a minimal threshold current density of 813 A/cm^2^. This generic concept can be applied to other material systems to provide higher integration density, more functionalities and lower total cost for photonics as well as microelectronics, MEMS, and many other applications.

## Silicon photonics: Building integrated lasers from the bottom up

A low-cost scheme for combining lasers with silicon photonic circuitry could make it more practical to move data efficiently using light pulses. Packaging standalone light-emitting chips, such as indium phosphide (InP)-based quantum well lasers, onto integrated silicon photonic microchips often has efficiency and reliability problems due to various factors from optical and mechanical perspectives. Yingtao Hu from Hewlett Packard Labs in Palo Alto, United States, and colleagues now demonstrate that InP-based quantum well lasers can be grown onto silicon waveguides by using a growth template. The team thermally joined a thin InP layer onto a patterned silicon substrate, and then epitaxially grew laser structures onto it. This process enabled fabrication of light-emitting crystals with low dislocation density on silicon for dense integration and scalable, low-cost manufacturing.

## Introduction

An integrated light source is considered to be the main challenge to realize all key photonic building blocks on Si. The mainstream commercial solution is to package a bulky III/V gain chip or finished laser die on a silicon (Si) photonic chip. A more favorable approach is to transfer III/V optical gain material on Si by wafer bonding, which allows low-loss evanescent optical coupling to Si photonic circuits and dense integration for enhanced chip performance and reduced packaging costs^1^. More recently, direct monolithic III/V epitaxy on Si has regained huge interest, and significant progress has been achieved^2–4^. Compared with the heterogeneous wafer-bonding approach, direct monolithic III/V growth on a 300-mm silicon wafer could lead to additional huge cost savings via cheaper substrates and wafer-scale III/V epitaxy. However, when directly growing III/V materials on Si substrates, the lattice mismatch, the difference in thermal expansion, and the different polarities of the materials result in a large number of crystalline defects known as dislocations on the order of 10^8^ cm^−2 5,6^. This large density of dislocations drastically degrades device performance and lifetime^7^. Recent tremendous progress with the demonstration of lasers with record efficiency^3^, a record threshold^4^, and a good lifetime^7^ is a result of newly developed novel substrate patterns^8^, intermediate buffer layers, and the use of defect-tolerant active regions, e.g., quantum dot (QD)^4^, on bulk Si substrates. However, several other critical issues associated with direct growth and monolithic integration with Si photonic circuits currently include potentially more challenges in heteroepitaxy on a silicon-on-insulator (SOI) substrate over bulk Si ones; difficulty in achieving efficient light coupling from the III/V active region to the Si waveguide due to inevitable several μm-thick buffer layers; and extra optical loss when light propagates in the dense dislocation zones. Therefore, we are motivated to develop a monolithic laser and photonic integration platform on SOI substrates with convenient optical coupling to Si waveguides, a low dislocation density, and a scalable cost-effective manufacturability.

In this paper, we demonstrate our recent development^9^ of a novel platform to integrate III/V materials into SOI substrates. It combines the advantages of monolithic growth and wafer bonding approaches, aiming to provide a low-dislocation-density, low-cost wafer-scale integration scheme. A combination of epitaxial growth and wafer bonding has been reported for lateral injection membrane lasers^10–12^ and double-heterostructure lasers^13^. However, the former requires a relatively complex and unconventional process to make p- and n-doping regions and to form a lateral p–i–n diode structure through an extra regrowth step. This unique process has enabled very impressive laser performance, but is not readily available in most commercial epitaxial growers. The latter uses a bonded thick InP growth template and buffer layer on a bulk Si substrate, which is challenging to integrate with other Si photonic circuits on the same chip, as discussed before. In this work, a standard ~2-μm-thick InGaAsP-based multi-quantum well (MQW) laser structure is epitaxially grown on a bonded InP-on-SOI template with significantly low threading-dislocation density (TDD), measured to be <1 × 10^5^ cm^−2^, despite the impact of thermal mismatch. Fabricated Fabry–Perot (FP) lasers show continuous wave (cw) and pulsed lasing up to 40 °C and a good threshold current and output power. Based on the excellent material quality of the regrown MQW and proof-of-concept laser performance, a number of merits of this demonstrated bonding plus epitaxy approach are discussed. The low dislocation density achieved in this integration approach would eliminate defect-induced lifetime concerns for practical applications. This bonding plus epitaxy idea is generically applicable for the integration of many more dissimilar materials. In particular, to compare with the presently popular III/V-to-Si wafer bonding approach, it enables III/V epitaxy on a much larger wafer scale at a lower epitaxy cost. Multiple sequential regrowths on the same bonded template allow integration of more than one III–V epitaxial structure for more advanced photonic integration, similar to bonding different III–V dies on the same Si wafer. However, our approach can lead to more intimate integration and benefits from a simpler single-bonding step.

## Results

**Fig. 1:**
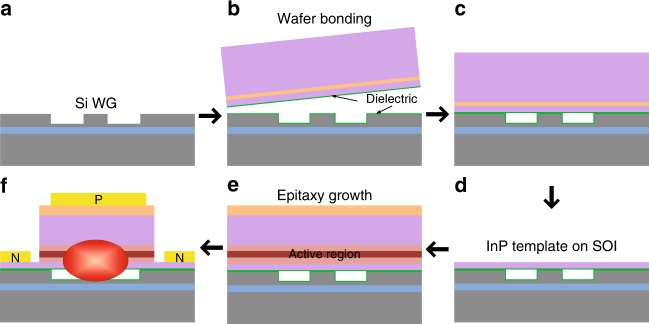
Schematic of the process of III/V-to-Si integration

**Fig. 2:**
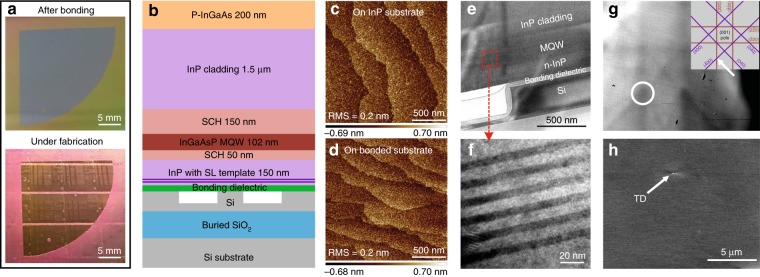
**a** Images of the bonded template and post-epitaxy fabrication wafer samples. **b** Epitaxial material structure on bonded InP-on-SOI substrate. AFM images of the epitaxy surface on **c** InP and **d** bonded substrates. **e**, **f** ECCI and **g**, **h** TEM images of epitaxy on the bonded substrate

Figure 1 shows a schematic of the process of our integration scheme. First, Si waveguides and some bonding-facilitating vertical outgassing structures^14^ were created in the SOI substrate (Fig. 1a). Second, an InP substrate wafer topped with a n-InP template layer and InGaAs etch-stop layer was bonded to the SOI wafer by direct wafer bonding (Fig. 1b, c). Upon selectively removing the InP substrate and InGaAs etch-stop layer to expose the n-InP template layer (Fig. 1d), the wafer-bonded InP-on-SOI substrate was loaded into a metal–organic chemical vapor deposition (MOCVD) chamber for epitaxial growth to form a conventional vertical p–i–n diode laser structure (Fig. 1e), similar to that previously reported for full III/V epitaxial stack bonding^15^. Finally, the heterogeneous wafer underwent a standard III/V process to fabricate laser devices (Fig. 1f). The bonded template was introduced to eliminate lattice and polarity mismatches between the Si and III/V material; thus, the thick intermediate buffer layer typically required in conventional III/V-on-Si direct epitaxy is not necessary. In addition, the n-InP template is only 150 -nm thick, similar to that previously reported in the full III/V stack bonding design. This enables facile light coupling from grown III/V active layers to Si waveguides. Figure 2a shows the heterogeneous wafer with the transferred n-InP template and the wafer under device fabrication after epitaxy. The heterogeneous wafer exhibited good yield and robustness after bonding annealing at 300 °C, epitaxy growth at 600 °C, and post-epitaxy device fabrication. Figure 2b shows the epitaxy structure grown on the bonded template, similar to well-demonstrated heterogeneous laser structures^16^ with low-loss evanescent coupling to the Si photonic circuits. The MQW is designed for light emission at 1.31 μm. The total epitaxy thickness is ~2 μm. An identical structure was grown on a witness InP wafer in the same growth chamber.

After epitaxy and before the post-epitaxy device fabrication, we conducted a detailed material characterization. Figure 2c, d is the atomic force microscopy (AFM) images of the epitaxy surface on InP and the bonded substrate, respectively, both showing an identical surface roughness with a root mean square (RMS) value of 0.2 nm. Figure 2e, f shows the cross-sectional transmission electron microscopy (TEM) images of the MQW epitaxy on the bonded InP-on-SOI substrate in the Si waveguide region. No defect was observed in a 10-μm-long and 0.1-μm-thick TEM specimen. MQW layers with good contrast and integrity are clearly exhibited in the high-magnification TEM image in Fig. 2f. Since we were unable to find any threading dislocation (TD) in the epitaxial material on the heterogeneous wafer from cross-sectional TEM imaging on much thicker (e.g., 0.75 μm) TEM specimens at various imaging conditions, we then performed plan-view TEM observations on a relatively large area of 30 × 12 μm^2^. Nevertheless, no TD but some misfit dislocations were observed with the plan-view TEM. Those misfits were observed at the interface between the upper SCH and InP cladding layer and within the InP cladding layer close to the interface by tilting cross-sectional TEM specimens. The existence of the misfits is explained as the result of thermal strain in the InP bonding template and the following epitaxy due to the difference in their thermal expansion coefficients and implies that the TDs are far away across the observed plan-view TEM area. Furthermore, we used electron-channeling contrast imaging (ECCI) to quantify the dislocation density in the plane view. Figure 2g shows the electron-channeling patterns corresponding to the three-beam (400) and (220) imaging conditions that were used. Figure 2h shows a representative image with only one TD. A total of 20 TDs were counted in 100 images with a total mapping area of 100 × 14.5 × 14.5 μm^2^. This led to a dislocation density of 9.5 × 10^4^ cm^−2^, only one order of magnitude higher than that on native InP substrates^17^ and two orders of magnitude lower than state-of-the-art conventional monolithic growth with a thick buffer layer^6^. Additional ECCI investigations observed small areas with more concentrated TDs, which were likely caused by bonding voids or dirt particles. We applied the same ECCI process to an InP witness sample for comparison, but could not see any TD due to a very low dislocation density for epitaxy on the native substrate.

**Fig. 3:**
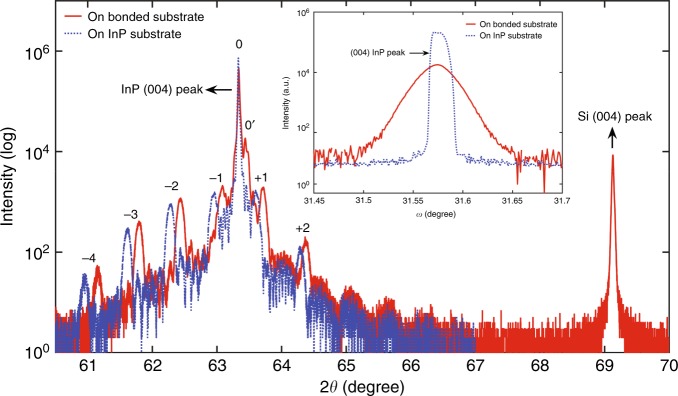
High-resolution XRD ω-2θ measurements of the epitaxy. Inset: high-resolution omega scans of the InP (004) peak

To further investigate the epitaxy structure on the bonded substrate, we conducted high-resolution X-ray diffraction (XRD) ω−2θ (rocking curve) measurements on the epitaxy samples. Figure 3 shows the XRD ω-2θ scan data for the InP substrate epitaxy and bonded samples. The scan range for the bonded sample was extended, so that the Si (400) peak from the SOI substrate could be collected as a reference. The Si (004) peak occurs at exactly 69.13°, which is the theoretical value, calibrating the 2θ value. The InP (004) main peaks (labeled as 0) for both curves are slightly shifted from the theoretical value, while the red curve shows a shifted InP (004) sub-peak (labeled as 0’). Our full material structure model fitting on ω-2θ revealed that the strongest InP (004) sharp peak is dominated mainly by the 1.5-μm InP cladding layer and that the shifted InP (004) sub-peak is fitted by introducing an ~1250-ppm compressive strain to the InP template layer. The strain-induced shift of the InP (004) sub-peak shifts the entire spectrum for the MQW on the bonded sample, as the MQW satellite peaks (−4, −3, −2, −1, + 1, and + 2) on the red curve are noticeably shifted toward higher angles than those on the blue curve. The XRD-fitted strain in the InP template layer is physically caused by the thermal mismatch between Si and InP. This strain is passed onto the subsequently grown MQW layers, leading to a shift in MQW satellite peaks. The aforementioned misfits likely relaxed the material at the location where it appears. As a result, the InP cladding layer appears to be largely strain-free, as evidenced by the un-shifted InP main peak. The MQW satellite peaks on both curves exhibit almost identical characteristic signatures, thus indicating good MQW structural similarity and integrity. In addition, full model fitting confirmed that the epitaxy structure from both samples matches the design. To probe the dislocation density of the epitaxy, high-resolution omega scans at fixed 2θ were measured on the InP (004) main peak for the epitaxy on both substrates (Fig. 3, inset). For the epitaxy on the InP substrate, the InP (004) peak contains contributions by the InP substrate and the 1.5-μm-thick InP cladding layer. Fitting with two Gaussian peaks revealed a peak with an intrinsic full-width at half maximum (FWHM) of ~16” attributed to the substrate and a broader peak with an FWHM of ~39.6” due to the cladding layer. The peak for the epitaxy on the bonded substrate is mainly contributed by the InP cladding layer since the InP template layer is much thinner, affecting its intensity contribution, and is shifted away. The FWHM is measured to be ~ 120”, which is three times larger than that on the InP substrate. We realized that the peak broadening is dominated by microstrain and specimen curvature. The broadening contribution of the angular rotation at the dislocation is too small to be used to calculate the exact dislocation density since XRD is suitable for the determination of the TDD in the range of 10^5^–10^9^ cm^−2^ ^18^. Although the increased InP (004) peak broadening for the epitaxy on the bonded substrate can be attributed primarily to the microstrain and increased curvature, to a lesser extent, the increased dislocation density may also make a contribution. This interpretation is consistent with our ECCI observations of one order of magnitude higher TDD in the epitaxy on the bonded substrate than that on the native InP substrate.

**Fig. 4:**
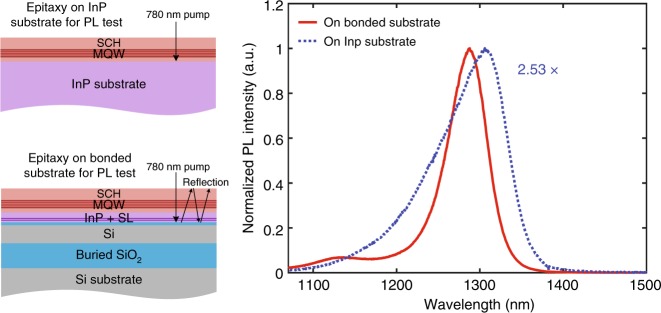
Schematic drawing of epitaxy structure and reflection of pumping light during PL measurements and the measured PL spectra for epitaxy on InP and the bonded substrate

Photoluminescence (PL) measurements were carried out at room temperature (RT) for the epitaxy on both InP and the bonded substrate after removing the p-InGaAs and p-InP cladding layers. During the measurements, the focus of the optical pumping laser with a wavelength of 780 nm was optimized by maximizing the MQW-peak PL intensity for each sample. We found that the measured PL intensity of the MQW from the bonded substrate sample was 2.53 times higher than that from the InP substrate sample, very similar to the 2.7 times higher PL intensity reported in reference^19^. This is mainly because strong reflections from the Si and buried-oxide layers couple with reflection from the top III/V surface to form a resonance cavity that enhances the PL pump efficiency in the Si substrate sample, as shown in the schematic drawing in Fig. 4. We plotted the normalized PL intensity distribution for the MQW in Fig. 4. It can be seen that the shape of the PL spectra is slightly different. The peak for the epitaxy on the bonded substrate is located at 1288.0 nm, and has an FWHM of 41.1 meV. The InP substrate is located at 1305.5 nm, and the FWHM is 61.9 meV. The residual thermal strain in the MQWs of the bonded substrate sample likely caused the differences in the PL profile and wavelength. In addition, a slight difference in the growth temperature on the top surface of the bonded substrate and on the InP substrate due to the difference in their thermal conductivity could cause composition changes, thus leading to PL shifts. Nevertheless, the epitaxy quality on the bonded substrate is comparable with that on the InP substrate.

**Fig. 5:**
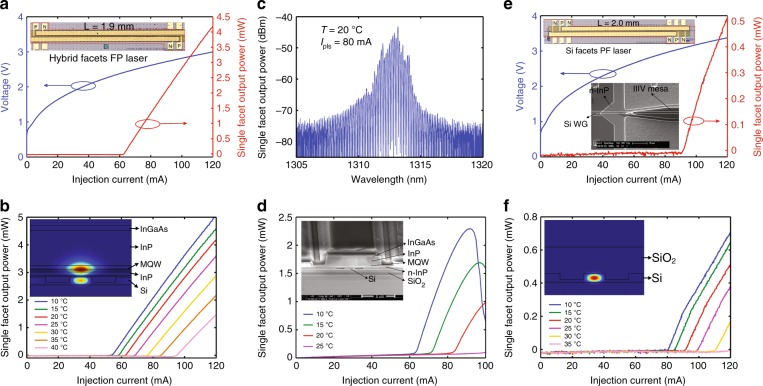
Lasers with a hybrid facet: **a** RT pulsed LIV (inset: microscope image of the device). **b** Pulsed LI up to 40 °C (inset: simulated mode profile at facets). **c** Device spectrum. **d** cw LI up to 20 °C (inset: SEM of the hybrid facet). Lasers with Si facets: **e** RT pulsed LIV (inset: microscope image of the device and SEM of the taper). **f** Pulsed LI up to 35 °C (inset: simulated mode profile at facets)

We then fabricated FP lasers, treating the Si substrate wafer as a conventional heterogeneous wafer, and applying the same fabrication procedure. We first characterized the fabricated FP lasers with diced and polished heterogeneous facets without coating, i.e., facets with a III/V mesa on the SOI waveguides; the fundamental mode profile is shown in Fig. 5b (inset). Figure 5a, b shows the light–current–voltage (LIV) curves at RT (20 °C) and LI curves up to a stage temperature of 40 °C, both of which are under the pulsed injection mode (0.5 μs, 0.25% duty cycle). The 1.9-mm-long device starts lasing at 61.8 mA and emits 4.2 mW from a single facet under a 120-mA current injection, corresponding to a reasonable threshold current density of 813 A/cm^2^ and an overall slope efficiency of 0.14 W/A. The observation of lasing at ~1313 nm under the pulsed mode at RT (Fig. 5c) matches our MQW design well. Figure 5d shows cw LI curves up to a stage temperature of 20 °C, with a slightly increased threshold due to device Joule heating. To prove convenient integration with other Si photonic circuits, we measured devices with two 50-μm-long III/V-to-Si taper structures. Si waveguide laser facets were formed by the same dicing and polishing procedure without coating. Figure 5e, f shows the LIV at RT and the LI curves up to a stage temperature of 35 °C under the same pulsed mode. The threshold current density of a 2.1-mm-long device with a 2.0-mm-long active region was calculated to be 1125 A/cm^2^. Due to the thin bonding template and unnecessary thick buffer layer, the output light from this laser can readily couple from the heterogeneous section to the Si waveguide section via III/V-to-Si tapers. The simulated fundamental output mode profile at the Si facet is shown in the inset of Fig. 5f. It is noted that we experienced some fabrication issues that significantly limited the device performance. First, the measured Zn doping concentration in the p-InP cladding was at least 10 × higher than that of the design, leading to a huge Zn optical absorption loss^20,21^. Second, devices suffered from an imperfect p-metallization process and a problematic wet-etching process when we attempted to selectively remove the InGaAsP MQW and expose the n-InP contact layer. The former resulted in a specific contact resistance of 1.29 × 10^–4^ Ω/cm^2^, two orders of magnitude higher than our normal value. The latter caused partial etching of the thin n-InP layer. Both contributed to the large serial resistance and slow turn-on in the diode, as indicated by the IV curves of Fig. 5a, e. Apparently, there is much room for improving the device performance by addressing the Zn doping error, metallization, and wet-etching issues. In addition, an improved taper design will minimize taper-induced extra cavity loss. Our current III/V-to-Si taper design consists of only the III/V taper with a linear shape and no taper on the Si waveguide. The coupling efficiency of this III/V-to-Si taper is 83% according to our simulations. The actual efficiency would be lower due to misalignment between the III/V taper and the underlying Si waveguide plus sidewall roughness in the fabricated devices. By introducing tapers into the Si waveguides and optimizing the shape of the tapers, we expect the coupling efficiency to increase up to 96% according to the simulations. Despite the growth and fabrication errors, this proof-of-concept demonstration paves a new way for large wafer-scale material and device heterogeneous integration with a number of merits, as discussed below.

## Discussion

The main advantage of conducting epitaxy on the bonded template is eliminating two out of three major dislocation root causes: lattice and polarity mismatches between the substrate (e.g., Si) and the function material (e.g., InP-based III/V) from epitaxial growth. The thermal mismatch between the substrate and template material would still cause dislocations in the regrown materials, but it is measured to be at a significantly low level. According to the aging tests of InAs QD lasers on Si near room temperature^7^, a reduction in the TDD from 10^8^ cm^−2^ to 10^6^ cm^−2^ can extend the laser lifetime from a few months to over 100 years. It is reasonable to expect that lasers from the regrowth on the bonded template with even lower dislocation density would eliminate the defect-induced lifetime concerns for all practical applications.

It is noted that the critical thickness of InP on a SiO_2_/Si substrate is calculated to be 200–430 nm from a conventional model^19,22^. The thickness from the interface of the bonding dielectric and InP bonding template to the observed misfit locations is ~450 nm. It is likely that the observed misfits are formed when the epitaxy thickness (including the InP template) reaches the critical thickness. Further study is expected to confirm this estimation.

**Fig. 6:**
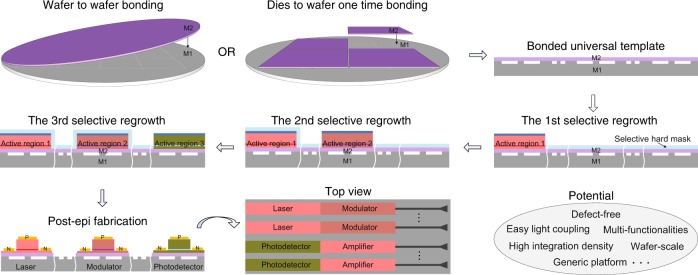
Schematic of the process of integrating lasers, amplifiers, modulators, and photodetectors with the bonding plus epitaxy platform

Epitaxy of this thickness with such a low dislocation density promises the possibility of growing many standard III/V structures for electronic, photonic, and MEMS applications. In addition, we believe that this bonding plus epitaxy approach is a generic method for many other heterogeneous material combinations. The substrate could be semiconductors, dielectrics, metals, etc., and the top grown material could be bulk materials, QWs, QDs, or other nanostructures. Sequential growth on the same template can be a routine procedure to enable advanced, large wafer-scale, dense photonic integration. A good example in silicon photonics is the integration of light sources, amplifiers, modulators, and detectors on a single chip with close proximity and low coupling loss by implementing multiple selective regrowth on a single-bonding template instead of bonding three or four types of epitaxial structures on each chip^11^. Figure 6 schematically shows an example of the process of integrating lasers^15^, amplifiers^23^, modulators^24^, and photodetectors^25^ onto the bonding plus epitaxy integration platform. The process begins with creating passive waveguide structures on a generic substrate wafer M1, e.g., Si. Then, a one-time bonding of M2, e.g., InP, onto M1, at either the wafer scale or chips-to-wafer scale, is executed to prepare the growth template. Necessary protection and sequential regrowth for the integration of three or four types of active devices are conducted. All regrown materials must be compatible with the template for low-defect growth. Advanced regrowth techniques such as butt-joint regrowth can be applied here to maximize the integration proximity and density and minimize reflection and other undesirable effects associated with abrupt topographic change. Since compound semiconductor substrates may account for significant wafer material costs, particularly for InP substrates, our solution provides the flexibility to reuse M2 substrates, particularly for wafer-scale template transfer^26^. Finally, device processing in sections of different functions in the same material system can share many fabrication steps towards seamless integrated chips.

Table 1 is a qualitative comparison of the production and operation costs of the same diode laser, built using different III/V-on-silicon integration approaches. All costs, including the substrate material (Si and InP substrate), III/V epitaxy, device fabrication (bonding and device fab), chip packaging, and operation, are compared separately among the four integration approaches. The marks (x, xx, xxx) indicate the relative cost level among the four integration approaches within each column, but do not indicate the cost differences over columns. Si does not participate in the operation of a standalone III/V diode laser; thus, the Si/SOI substrate cost is zero for the finished III/V diode laser chip packaged on Si. The Si/SOI substrate cost is similar for the other three approaches since they are all able to reach sizes up to 12 inches. The additional epitaxy step to prepare a thin III/V template layer, which is unique in our bonding plus epitaxy approach, incurs marginal cost due to the short and simple growth. Our approach has the flexibility to reuse the III/V substrate^26^, which can further offset the additional cost from this additional template epitaxy. Therefore, the III/V substrate cost is similar for the three approaches using the III/V substrate. Considering that a 12-inch carrier in an epitaxy reactor could accommodate 24, 10, and 7 pieces of 2-, 3- and 4-inch substrate wafers, respectively, one can easily determine that the respective effective epitaxy areas for these three size wafers are 49, 54, and 72% of the full 12-inch carrier area. As a result, the III/V epitaxy cost is relatively high for the first and second approaches in the table since they are still limited to complex laser structure epitaxy on 2–4 inches of substrate, particularly for InP. The III/V epitaxy cost of our bonding plus epitaxy approach is even lower than that of the direct epitaxy on Si approach because thick buffer layers are not necessary here, which reduces the amount of epitaxy time and source material needed. The bonding cost is similar for the wafer bonding approach and the bonding plus epitaxy approach if only one type of III/V epitaxial structure is used. However, in the scenario of multiple III/V epitaxial structure integrations on the same chip, with multiple small III/V dies bonded onto each Si photonic chip, hundreds of III/V dies per Si wafer are certainly more complicated and expensive, with potentially lower yield than just bonding one large III/V die in each Si photonic die. Large wafer-scale chip fabrication can tremendously reduce the manufacturing costs associated with unit devices; thus, the 12-inch process should be the most cost-effective in this category. For the finished III/V chip packaged with the Si approach and the direct III/V-epitaxy-on-Si approach, the high packaging cost is due to the high-accuracy alignment needed to couple the light from a separated diode laser chip to Si photonic components. Unavoidable modal mismatch loss from this chip-to-chip optical coupling requires higher laser output power, a subsequently higher energy bill in operation and a potentially shorter lifetime. Therefore, the bonding plus epitaxy approach can be very cost competitive overall. Its high integration level and density minimizes the chip size, packaging effort, and link power budget. This allows the lasers and whole system to operate in an energy-efficient regime.

**Table 1:** Qualitative comparison of production and operation costs of the same diode laser built using different III/V-on-silicon integration approaches

Unit laser area cost	Substrate material		III/V epitaxy	Fabrication		Packaging	Operation (energy $)
Approach	Si/SOI (12 inch)	InP		Bonding	Device fab		
Finished III/V chip packaged with Si^27^	None	xxx (2–4 inch)	xxx (2–4 inch)	None	xxx (3 inch)	xxx	xx
III/V wafer bonding on Si^28^	x	xxx (2–4 inch)	xxx (2–4 inch)	xx	x (12 inch)	x	x
III/V epitaxy on Si^2–4^	x	None	xx (12 inch)	None	x (12 inch)	xxx	xx
Wafer bonding plus epitaxy	x	xxx (2–4 inch, template epitaxy included)	x (12 inch)	x	x (12 inch)	x	x

*x* low, *xx* medium, *xxx* high

In conclusion, we have proposed and demonstrated a special III/V-on-Si photonic integration platform formed by combining the benefits of both methods. We demonstrated monolithically integrated 2-μm-thick InP-based MQW laser epitaxy with a standard vertical p–i–n diode structure on the bonded InP-on-SOI substrate. The regrown epitaxy shows high material quality with significantly low dislocation density. Successful pulsed and cw lasing with good threshold current density and output power are achieved, despite incorrectly high p-type doping and fabrication imperfection. We emphasize that the bonding plus epitaxy approach is a general approach for combining different materials onto various substrates. Compared with other integration schemes, the method can be potentially cost-competitive and highly scalable, with a high integration proximity and density. By combining traditional heterogeneous and monolithic photonic integration^11,13^, we can achieve large wafer-scale μm-thick III/V epitaxy and advanced all-in-one photonic integration for a variety of applications.

## Materials and methods

The fabrication of the III/V-on-Si MQW lasers began with a 4-inch SOI wafer with a 350-nm-thick top Si layer and a 1-μm-thick buried-oxide layer. An i-line stepper and a reactive-ion etcher were used to define the Si waveguides and the vertical outgassing channels on the SOI wafer^14^. A quarter cleaved from the patterned 4-inch SOI wafer was used to bond with a quarter of 2-inch InP-based growth template wafer. The InP-based template wafer includes a 150-nm-thick n-InP layer and a 200-nm-thick InGaAs layer as a wet etch stop layer on the InP substrate. After thorough wafer cleaning in solvent separately, the patterned SOI wafer and the InP-based template wafer underwent a 7-nm-thick SiO_2_ deposition onto their top surfaces in an atomic layer deposition (ALD) system simultaneously. The InP wafer was then bonded onto the SOI wafer manually, followed by press holding in a bonding fixture at an annealing temperature of 300 °C for 2 h. Upon InP substrate removal by mechanical polishing and thereafter selective wet etching in a solution of HCl:H_2_O = 3:1, a InP growth template layer protected with the InGaAs wet etch stop layer was formed on the SOI substrate. Then, the vertical outgassing channels were opened up by etching through the n-InP and InGaAs layers by using i-line lithography and dry etching. The bonded wafer was further sawed to fit the 2-inch substrate carriers in the growth chamber that would be used. The InGaAs etch stop layer was selectively wet etched in a solution of H_3_PO_4_:H_2_O_2_:H_2_O = 1:1:38 right before loading the bonded wafer into a MOCVD chamber to epitaxial growth on a fresh InP surface. The growth was carried out at a substrate temperature of 600 °C. The epitaxial structure in the sequence of growth consists of a first separate confinement heterostructure (SCH) layer (50 nm), six periods of InGaAsP QWs (total of 102 nm), a second SCH layer (150 nm), a p-InP cladding layer (1.5 μm), and a p-InGaAs contact layer (200 nm). After the growth, the heterogeneous wafer was treated as a normal wafer and underwent a standard III/V process flow to form ridge laser devices. Prior to the formation of mesas, protons (H + ) were implanted on the two sides of each mesa to define a ~ 4-μm-wide p-type current channel in the mesa. P-contacts with Pd/Ti/Pd/Au/Pd/Ti (3/17/17/403/10/10 nm) alloy stack were formed in the center of mesas by a lift-off process. Then 8-μm-wide mesas were created using i-line photolithography and CH_4_/H_2_/Ar-based plasma reactive ion etching through the p-type layers, followed by selective wet etching of the MQW layers to the n-type layers in a solution of H_3_PO_4_:H_2_O_2_:H_2_O = 1:1:38. N-contacts with Pd/Ge/Pd/Au/Ti (10/110/25/1000/10 nm) alloy stack were created onto the exposed n-type InP layer in both side of mesas and 2 µm away from the edge of mesas. A ~800-nm-thick SiO_2_ passivation layer was then deposited onto the wafer, followed by via openings on p- and n-contact regions. A 1-μm-thick Au was deposited and lifted-off to form the electrode metal pads. Upon the completion of the device fabrication, the wafer was diced into bars according to the columns of devices and waveguide facets were polished to forming 1.9 mm or 2.1 mm-long laser cavities.

The device bar was mounted on a copper stage with a thermo-electric cooling (TEC) chip, and the lasers were driven by electrical current injections through the p-electrode and n-electrode metal pads. The laser output from one waveguide facet is collected by a lensed single-mode fiber and then characterized by using a photodetector or optical spectral analyzer.
